# Public health nurses’ experiences of ethical responsibility: A meta-ethnography

**DOI:** 10.1177/09697330231209294

**Published:** 2023-10-27

**Authors:** Anne Clancy, Julia Thuve Hovden, Runa Anneli Andersen, Hilde Laholt

**Affiliations:** Department of Health and Care Sciences, Faculty of Health Sciences, 60482 UiT The Arctic University of Norway, Tromsø, Norway

**Keywords:** Ethical responsibility, moral responsibility, meta-synthesis, meta-ethnography, public health nurses

## Abstract

Public health nursing is grounded in public health ideologies and fundamental nursing values. Researchers have argued that ethical responsibility from the perspective of the nurse is an understudied phenomenon. This meta-ethnography provides in-depth knowledge of how public health nurses (PHNs) experience ethical responsibility when working to prevent injury and disease, and promote health and well-being in children, young people and their families. There are reciprocal findings across the 10 included studies. The findings reveal that these nurses often feel alone, have worries and uncertainties and are afraid of doing wrong. They describe unclear boundaries in their work, creating a heightened sense of responsibility. PHNs fight lonely battles. Yet they show courage and commitment and are ready to stand up and fight for children and families who do not receive adequate care. A line of argument is developed and the metaphorical phrase Chivalrous knights in moral armour is used to express the authors’ overall interpretations of the findings. Reflection on the findings shows how the different dimensions of ethical responsibility are interconnected. The nurses’ ethical sensitivity enables them to feel compassion for others and they show indignation when vulnerable others are not treated with dignity and respect. Indignation and compassion are interrelated, and when human life and dignity are threatened, the ethical demand to respond emerges. Indignation is a precursor to moral courage, and the nurses’ moral sensitivity and respect for their clients emboldens them to stand up for vulnerable others. The findings also illustrate the paradoxical nature of freedom. Freedom of choice due to unclear boundaries heightens the nurses' sense of responsibility. This research is an important step in theory development and has implications for further research, education and practice.

## Introduction

This article deals with public health nurses’ (PHNs) experiences of ethical responsibility. Public health nursing is grounded in public health ideologies and fundamental nursing values. Public health involves promoting the health and well-being of populations. The nurses’ code of ethics guides PHNs in working in a morally responsible way.^1^ Respect for each person’s inherent humanness, dignity and vulnerability is a core nursing value.^2^ These values are independent of cultural norms or position in society and are influenced by personal morals, culture, work experience and context.^3^ Tensions exist between expectations, personal values and professional regulations in nursing practice.^4^ A code of ethics describes professional obligations but does not provide nurses with insight on situational ethical responses at a relational level. Moral responsibility requires a response to the ethical demand from another person. In this sense, the terms ‘ethical’ and ‘moral’ can be used to describe the process where the nurse, as a responsive carer, must choose between different alternatives and act correctly in a situation.^5^ This process is understood as a relational way of being, which emanates from one’s inner self, created through a dialectic process with others.^6^ The authors use the term ethical responsibility to describe ethical and moral challenges the nurses’ face.

Public health nursing has been described as an autonomous profession with unclear boundaries and responsibilities.^7,8^ PHNs provide universal services and are in a unique position to prevent injury and disease, and promote health and well-being in children, young people and families. The philosophy of public health nursing is based on the belief that care for the individual, family and groups enhances population health.^9^ PHNs’ role in public health and their strategic front-line position in a constantly changing practice can increase their feelings of responsibility and involvement in morally challenging situations.^10^ PHNs experience moral dilemmas where there is no ideal solution to a problem or when they feel ethically obliged to perform two nursing actions but cannot do both.^11^ These morally challenging situations can arise when ethical values clash and the nurse must prioritize. The situation can entail, for example, respecting a person’s autonomy while also feeling responsible for preventing harm.

### Philosophical perspectives on ethical responsibility

Levinas’^12,13^ philosophy is an ethics of responsibility relevant to the study of ethical responsibility in public health nursing. Responsibility involves responding to the call of another person; it is something I am given but cannot contain. Levinas does not see ethical responsibility as a solid fundament, but more an infinite abyss that transcends the boundaries of time, space, language and knowledge. As Clancy^7^ writes:

Even though Levinas writes about intersubjective dependency, his concept of alterity recognizes each person’s separateness, existential loneliness and humanness. Recognition of the seemingly contradictory forces of dependency and separateness is relevant in the study of relationships between PHNs and the people they meet in their work.^7^

Løgstrup and Fink^14^ write that we are responsible when something or someone depends on us and if we fail in our duty, we are guilty of not acting ethically. Levinas^12,15^ writes that our responsibility for others can never deprive others of their own responsibility.

Wallinvirta^16^ examined the inner meaning of responsibility in nursing. Her doctoral thesis has an etymological approach within a caring science tradition and is based on the philosophies of Buber, Levinas and Kierkegaard. The author carried out an integrative review and meta-synthesis of 60 quantitative and qualitative articles related to responsibility in different nursing settings. The review was limited to articles published in the Nursing Ethics journal between 2004 and 2008. Wallinvirta’s approach resulted in the thesis that the core of responsibility comprises freedom, guilt and love. Clancy and Svensson^17^ studied the phenomenon of ethical responsibility in public health nursing. Levinasian ethics was used as a philosophical framework for interpreting empirical data collected from experienced PHNs. The in-depth philosophical analysis resulted in the presentation of five dimensions of ethical responsibility in public health nursing: personal responsibility; care, worry and uncertainty; being alone; boundaries; satisfaction and temporality.

The ethics of responsibility emerges from reflection on the lived experiences of PHNs who express the ethical demands in their work.^17^ Few primary research studies have focused on moral or ethical responsibility as experienced by PHNs.^17,18^ Extensive searches have not revealed systematic reviews or meta-syntheses of PHNs’ experiences in this regard.

### Aim

The aim of this study is to conduct a systematic literature review and meta-ethnography that explores PHNs’ experiences of ethical responsibility. This meta-ethnography can raise awareness of ethical responsibility in public health nursing and provide insight into the challenges these nurses face. The synthesized research findings may have implications for education and practice and aid theory development in the field.

## Method

Meta-ethnography is the chosen approach. The meta-ethnographical approach^19,20^ can deepen our understanding of a phenomenon and promote the development of new theories, concepts and models.^21^ This method can be more informative than a broad sampling strategy that provides a superficial understanding of a phenomenon.^20,22^ The aim of a meta-ethnography is to synthesize the results of qualitative studies to create a deeper understanding. We followed the eMerge guidelines for reporting a meta-ethnography,^21^ which provided guidance on writing up the study. The authors also provide a meta-summary^23^ of the characteristics of the included studies and the distribution of findings.

### Data collection

A purposeful sampling strategy was used. This search strategy is an iterative process that must be transparent, and the review question and central concepts must be presented in a logical way. The SPIDER diagram (Table 1) helped clarify the research focus, present the key search terms and phenomenon of interest,^24^ which was: PHNs’ experiences of ethical responsibility. The search terms were based on the objectives of the research. These were developed with a head librarian to refine the inclusion and exclusion criteria.

**Table 1.** SPIDER (Sample, Phenomenon, Interest, Design, Evaluation, Research) diagram.

Sample	Phenomenon	Interest	Design	Evaluation	Research type
Public health nurses, school nurses, health visitors, community health nurses	Ethical or moral responsibility	Experiences of ethical and moral responsibility	Qualitative studies/data with rich textual descriptions by PHNs	Public health nurses’ perceptions and experiences	Qualitative data, rich descriptions, individual and focus group interviews
	**Search terms**	**Search terms**	**Search term**	**Search term**	**Search term**
‘Public health nurs*’ OR ‘health visitor*’ OR (‘MH community health Nursing+’) OR ‘school nurs’*	AND (ethic* or moral) AND responsib*	AND (ethic* or moral) AND responsib*	Qualitative	View * OR experience* OR perceptions* OR attitude* OR particpat* OR opinion	Interview OR/AND focus groups

We included qualitative research articles in English from any publication year on PHNs' experiences of moral or ethical responsibility (Table 2). Searches were conducted in CINAHL, MEDLINE and Web of Science in March 2022 by the first and fourth authors in collaboration with a head librarian. Subject terms and free-text words were combined with Boolean operators. All four authors performed manual article searches and forward citation reference searches in Google Scholar in April 2022 and backward searches in reference lists of relevant articles.

**Table 2.** Inclusion and exclusion criteria.

Inclusion	Exclusion
• Research articles that portray PHNs' experiences of ethical or moral responsibility in preventative and health promotion work with children and young people (0–20 years) and their families	• Studies that include PHNs working with older people/life-span perspectives
• Primary research articles in English	• Studies using quantitative methodology
• Studies using qualitative methodology, individual and focus group interviews, and free-text survey data	• Reviews and meta-syntheses that do not represent the first-person perspective
• Any year of publication	

**Figure 1.**
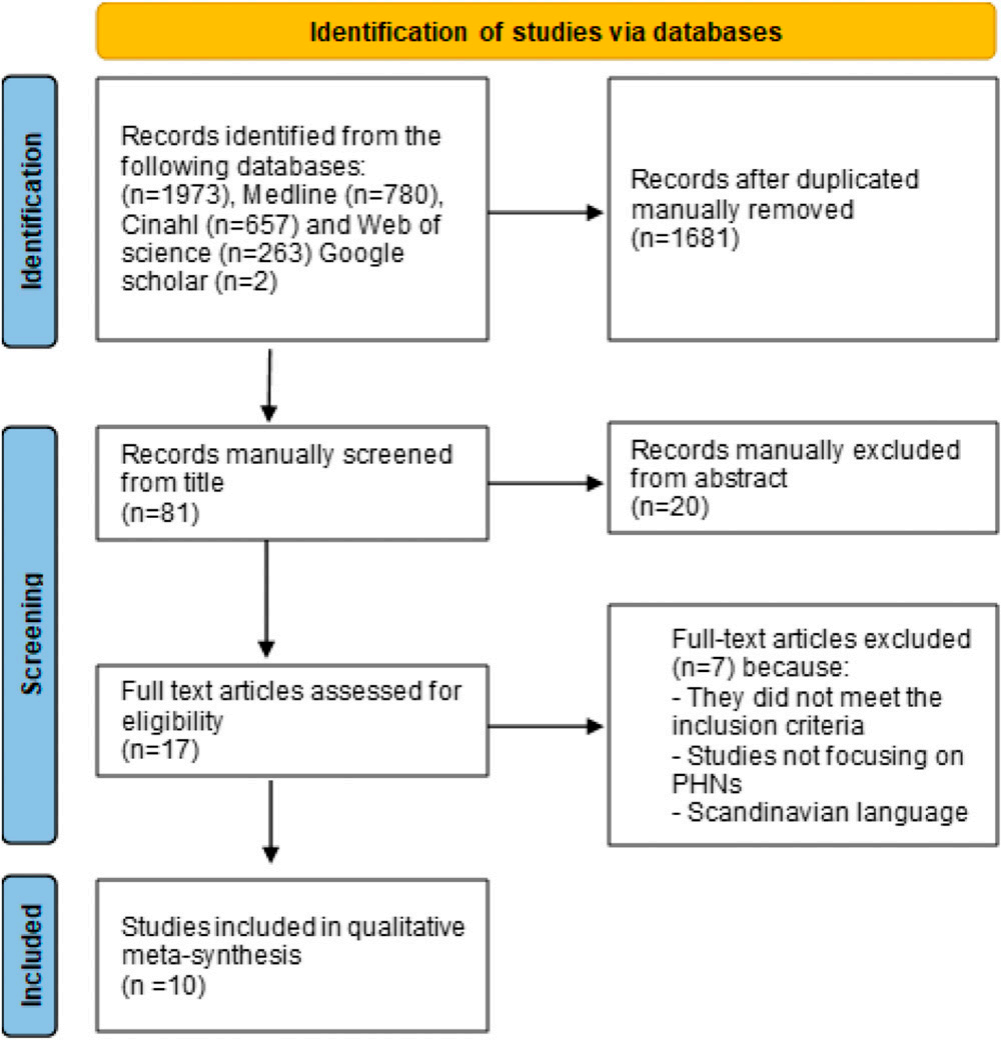
Flow chart inclusion and exclusion process.^25^

A PRISMA flow diagram^25^ (Figure 1) illustrates the inclusion and exclusion process. The systematic search resulted in 1973 articles.

### Screening and selection of studies

The primary screening process and selection of studies was as follows. The first and fourth authors transferred titles with a digital link to the published article to a Word document. Duplicates were then removed. Based on the exclusion and inclusion criteria, all titles and abstracts were screened. In the third phase, all authors examined full texts of potentially relevant studies. Issues of contention were discussed, and articles were not excluded until consensus was reached. This process took place between October 2022 and January 2023 and resulted in the inclusion of 10 studies (Figure 1; Table 3). The authors had regular online and face-to-face meetings to discuss all stages of the analysis. Articles were read independently and in pairs, and original studies written by the authors of this meta-ethnography were discussed by other team members. The four authors then individually reviewed the 10 included articles and placed relevant findings into a table (supplementary material). The team discussed how to synthesize the findings in the next phase.

**Table 3.** Characteristics of the included studies.

Number	Author, year	Country	Title	Aim	Design/method	Setting and sample	Major findings	Journal
**1**	Clancy and Svensson ^17^ 2007	Norway	‘Faced’ with responsibility. Levinasian ethics and the challenges of responsibility in Norwegian public health nursing	To explore the phenomenon based on the ethics of responsibility as reflected upon by the philosopher emmanuel Levinas (1906-1995)	Qualitative individual interviews, hermeneutic phenomenology of Max van Manen	Urban/rural/setting: Sample *n* = 5 PHNs working in child health clinics and schools	Personal responsibility, boundaries, temporality, worry, fear and uncertainty, being alone, a sense of satisfaction	*Nursing Philosophy*
**2**	Clausson et al.^29^ 2015	Sweden	Challenges of documenting schoolchildren’s psychosocial health: A qualitative study	To explore school nurses’ experience of challenges in documenting schoolchildren’s psychosocial health	Qualitative design, focus group discussions and qualitative content analysis	School health services Sample: Six groups with *n* = 33 school nurses	One overarching theme: Having to do one’s duty and being afraid of doing wrong. Three sub-themes. Uncertainty related to the nurse’s own ability, concerns about future consequences, and strategies for handling documentation	*The Journal of School Nursing*
**3**	Dahl et al.^32^ 2014	Norway	The meaning of ethically charged encounters and their possible influence on professional identity in Norwegian public health nursing: a Phenomenological hermeneutic study	To illuminate PHNs’ experiences of ethically charged encounters and to reflect upon how these experiences can influence professional identity	Qualitative individual interviews interpreted with a phenomenological hermeneutic method inspired by the philosophy of Paul Ricoeur	Urban/rural small, middle and large communities in two counties. Health clinics and school health services. Sample: *n* = 23 PHNs	Four themes: feeling responsible, being committed, feeling confident and feeling inadequate	*Scandinavian Journal of Caring Sciences*
**4**	Duncan^33^ 1992	Canada	Ethical challenges in community health nursing	To begin to describe the nature of ethical conflicts in community health nursing	Survey with qualitative free-text data	Community setting, urban and rural. Sample: *n* = 30 community health nurses	Situations involving high-risk parenting provided most serious ethical challenges. Strategies to help nurses caring for such vulnerable clients are described, implications for community health nursing practice and nursing education in light of current changes and challenges	*Journal of Advanced Nursing*
**5**	Heggestad al.^30^ 2021	Norway	Ethics reflection groups for school nurses	To explore how school nurses experience their role and their participation in ethics reflection groups, using a model for systematic ethics reflection, the centre for medical ethics model	Qualitative design, focus group interviews and thematic analysis	School health services, Sample: *n* = 6 psychologists and *n* = 6 school nurses	The demanding role of a school nurse, challenges with ethics reflection groups, advantages of using the centre for medical ethics model in ethics reflection	*Nursing Ethics*
6	Hilli, and Pedersen ^36^ 2021	Sweden	School nurses’ engagement and care ethics in promoting adolescents’ health	To describe care ethics in the context of school nurses’ health-promoting activities among adolescents in secondary school	Explorative descriptive methodology with qualitative individual semi-structured interviews. Content analysis	School health services, *n* = 8 school nurses in western Sweden	Three main categories with sub-categories: Engagement and caring for adolescents, collaboration and involvement of important stakeholders, and strong commitment to promoting adolescent health	*Nursing Ethics*
**7**	Kvamme, and Voldner, ^28^ 2021	Norway	Public health nurses’ encounters with undocumented migrant mothers and children	To describe how public health nurses experienced challenges and dilemmas in ensuring the best interest of undocumented migrant children	Qualitative descriptive design. Focus group interviews and semi-structured individual interviews. Qualitative content analysis	Four child health centres, *n* = 7 PHNs	Three main themes were identified: Building trust, ensuring the child’s best interest, and dilemmas and challenges in ensuring the child’s best interest	*Public Health Nursing*
**8**	Laholt et al.^31^ 2019	Norway	Ethical challenges experienced by public health nurses related to adolescents’ use of visual technologies	To explore how school nurses identify and resolve ethical challenges involved in the use of visual technologies in health dialogues with adolescents	Qualitative study. Focus group discussions. Analysis using systematic text condensation	School health services. Seven groups of *n* = 40 PHNs	Situations that raised ethical issues, identifying and navigating ethical challenges and resolving them through peer dialogue	*Nursing Ethics*
**9**	Oberle and Tenove^34^ 2000	Canada	Ethical issues in Public health nursing	To explore ethical issues in public health nursing	Exploratory descriptive study involving qualitative individual interviews. Thematic analysis, by Corbin and Strauss	Twenty-two PHNs working in public health nursing centres, *n* = 11 in rural and *n* = 11 in urban settings	Analysis revealed five interrelated themes, relationships with other clinicians, system issues, character of relationships, respect for people and putting oneself at risk. All aspects of public health nursing have ethical components	*Nursing Ethics*
**10**	Tollefsen et al.^18^ 2021	Norway	Nurses’ experiences of ethical responsibility: A hermeneutic phenomenological design	To explore how intensive care and public health nurses experience responsibility	Qualitative design, individual interviews. Hermeneutic phenomenological analysis	Sample *n* = 5 intensive care nurses and *n* = 5 PHNs. Setting: The PHNs worked in small rural municipalities	Four themes: feeling alone, feeling worried and uncertain, a sense of satisfaction, a personal commitment	*The Nordic Journal of Nursing Research*

### Contextual information

Contextual information illustrates how the included studies are related.^21^ Table 3 provides contextual information and characteristics of the included studies.

We registered the study in the PROSPERO international prospective register of systematic reviews, ID:389190.

### Analysis

#### The analysis process: synthesis and meta-synthesis

The authors conducted a meta-synthesis of research studies on ethical and moral responsibility based on a meta-ethnographical approach. Meta-ethnography^20^ is a seven-step approach that includes the following phases: Getting started, deciding what is relevant to the phenomenon of interest, reading the studies, deciding how they are related before translating the studies into one another, then creating and finally expressing the synthesis. This approach allows for an insightful, novel interpretation of findings in the primary studies and has the potential to develop a theoretical understanding of the phenomenon. In this approach, special attention is given to metaphors and meaningful phrases.^20^

The goal of a meta-ethnography is to explore the phenomenon of interest by reaching conceptual clarity.^26^ The CASP quality assessment tool (Table 4) was used to assess the rigour, relevance and quality of each study. According to Sattar et al.,^27^ the CASP instrument is useful for identifying the strengths and weaknesses of each study. However, the final inclusion of studies was based on their relevance and depth of description and guided by the authors’ comprehensive knowledge of public health nursing. According to Sandelowski and Barroso,^23^ a study can be relevant for inclusion despite not meeting all the criteria for quality inclusion.

**Table 4.** CASP checklist for appraising qualitative studies.

Number	Study	Was there a clear statement of the aims of the research?	Is a qualitative methodology appropriate?	Was the research design appropriate to address the aims of the research?	Was the recruitment strategy appropriate to the aims of the study?	Was the data collected in a way that addressed the research issue?	Has the relationship between researcher and participants been adequately considered?	Have ethical issues been taken into consideration?	Was the data analysis sufficiently rigorous?	Is there a clear statement of findings?	How valuable is the research?
1	Clancy and Svensson ^17^	Y	Y	Y	Y	Y	CT	Y	Y	Y	Valuable
2	Clausson et al^29^	Y	Y	Y	Y	Y	CT	Y	Y	Y	Valuable
3	Dahl et al^32^	Y	Y	Y	Y	Y	CT/Y	Y	Y	Y	Valuable
4	Duncan^33^	Y	Y	Y	Y	Y	CT	Y	Y	Y	Valuable
5	Heggestad et al^30^	Y	Y	Y	Y	Y	Y	Y	Y	Y	Valuable
6	Hilli and Pedersen ^35^	Y	Y	Y	Y	Y	CT	Y	Y	Y	Valuable
7	Kvamme and Voldner ^28^	Y	Y	Y	Y	Y	Y	Y	Y	Y	Valuable
8	Laholt et al^31^	Y	Y	Y	Y	Y	Y	Y	Y	Y	Valuable
9	Oberle and Tenove^34^	Y	Y	Y	Y	Y	Y	Y	Y	Y	Valuable
10	Tollefsen et al^18^	Y	Y	Y	Y	Y	Y	Y	Y	Y	Valuable

Y = yes, *N* = no, CT = can’t tell.

From: Critical Appraisal Skills Programme (reference). CASP-Qualitative-Checklist-2018.pdf.

#### Synthesizing the translations and performing the line of argument synthesis

##### The findings from each of the 10 studies were read carefully by all authors

Special attention was paid to metaphors and meaningful phrases cited by the participating PHNs in the primary studies. Metaphors and concepts used by the PHNs evoked vivid images that gave a broader understanding of the nurses’ experiences. These are presented as primary quotes in the findings section. Second order concepts and metaphors created by the authors of the primary studies and reported in the findings were also noted. The discussion sections were then read for possible additional second order concepts. The authors' interpretation of relevant and illustrative concepts, phrases and metaphors were recorded for each study before being compared and contrasted across studies.

**Figure 2.**
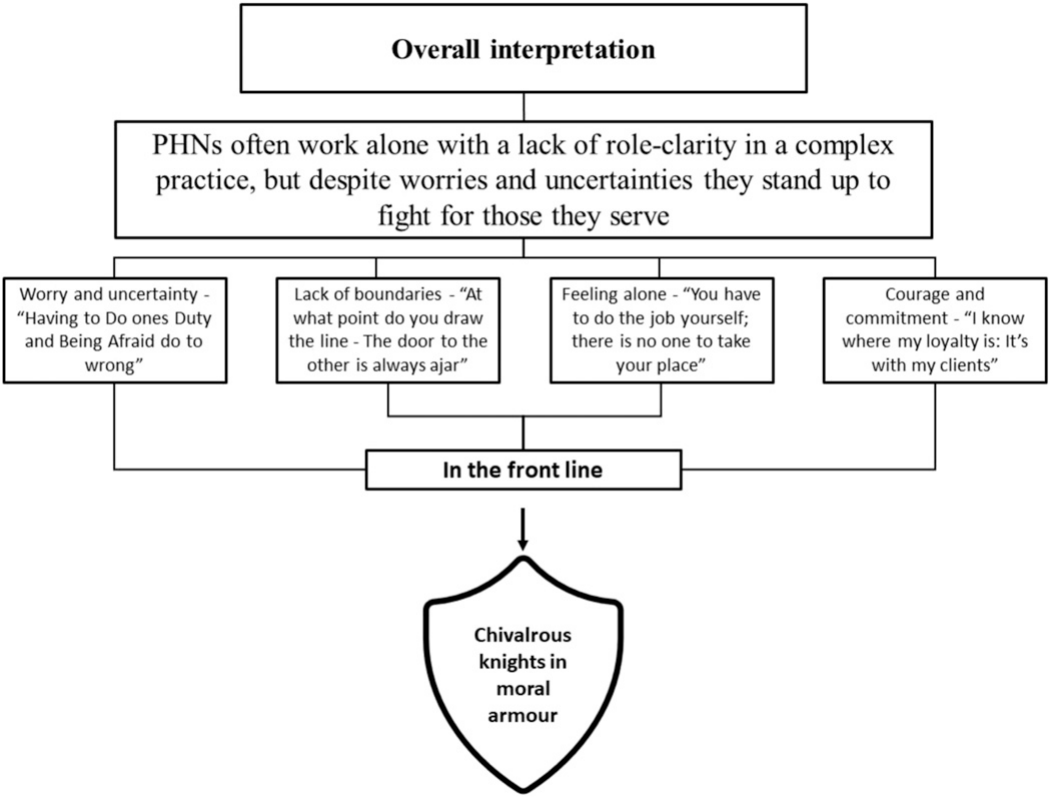
Overview of the authors’ overall interpretation.

According to Noblit and Hare,^20^ a synthesis requires creating a more comprehensive understanding of a phenomenon where the results of the synthesis will be greater than the sum of the parts. Noblit and Hare^20^ describe the process of synthesizing the studies as the translation of studies into one another, where similarities in concepts and metaphors are noted (reciprocal translation) and differences explored (refutational translation). A translation process of comparison of similarities and differences was carried out by all four authors, individually, in pairs and collectively. This process was continued until all articles were translated into each other, and common themes emerged. Common themes across studies were grouped together into four main themes. Figure 2 illustrates the authors’ overall interpretation. A line of argument^20^ was developed. This is presented as an overarching metaphor that provides a new and more comprehensive understanding of ethical responsibility in public health nursing.

The meta-summary^23^ gives the reader a summative overview of the characteristics of the included studies.

## Findings

The findings were similar across studies and the authors found no refutational results. The line of argument is expressed in the overriding metaphor ‘Chivalrous knights in moral armour’ (Figure 2). The meta-summary (Table 5) illustrates a high frequency of key findings across studies.

**Table 5.** Meta-summary of characteristics of included studies.

Item	Study number (As shown in Table 3)	No. of studies reporting each item (*N* = 10)
**Community setting**		
Urban and rural	1,2,3,9,10	5
School health services	2,5,6,8	4
Child health centres	7	1
**Description of sample**		
PHNs working in child health centres combined with school health services	1,3,9,10	4
PHNs working in child health centres with migrant mothers and children	7	1
PHNs working in school health services	2,5,6,8	4
Community health nurses in prevention programmes for families	4	1
**Number of participants in total**		145
**Ethical focus and challenges related to ethical responsibility**		
Aspects of public health nursing role	1,3,4,5,6,9,10	7
Visual technologies	8	1
Documentation of pupils’ health	2	1
Work with migrant families	7	1
**Theoretical framework described ethical theory**		
Caritative caring (Eriksson K. & Watson, J)	6	1
Ethical responsibility (Levinas, E.)	1,3,10	3
The ethics of care (Mol, A.)	8	1
No specific theory	4,2,5,7,9	5
**Data collection method**		
Individual interviews	1,3,6,9,10	5
Focus group interviews	2,5,8	3
Both individual and focus group interviews	7	1
Survey with free-text descriptions	4	1
**Frequency of findings**		
Worry and uncertainty	1,2,3,4,5,7,8,9,10	9
Lack of boundaries	1,2,3,4,5,6,7,8,9,10	10
Feeling alone	1,2,3,4,5,6,7,8,9,10	10
Courage and commitment	1,2,3,4,6,7,8,9,10	9
**Participants in analysis process**		
Research team of two or more	2,5,6,7,8,9	6
Not described	1,3,4,10	4
**Authors background**		
Public health nurses	1,2,3,4,6,7,8,10	7
**Other authors**		
Registered nurse	5	1
Medical doctor (GP)	5	1
Sociologists	1,3,8	3
Philosopher	8	1
Not described	9	1

The synthesis process resulted in the following four main themes:

Worry and uncertainty *-‘Having to do one’s duty and being afraid to do wrong*', Lack of boundaries - ‘*At what point do you draw the line?*', Feeling alone - ‘*You have to do the job yourself; there is no one to take your place*', and Courage and commitment *-* ‘*I know where my loyalty is: it’s with my clients*'.

The findings provide a more comprehensive understanding of the phenomenon and the metaphorical phrase ‘*Chivalrous knights in moral armour*’ is used to express the authors’ overall interpretations of the findings. The nurses’ experiences of moral responsibility show us that despite a time span of 30 years there are reciprocal findings across studies.

### Worry and uncertainty – ‘Having to do one’s duty and being afraid to do wrong’

PHNs spoke of worry and uncertainty regarding the dilemmas they faced, not knowing what to do and being afraid of doing wrong.^17,28-32^ They described responsibility as a duty to deal with or take care of individuals and communities.^17^ Experiencing ethical responsibility for others and deciding how to respond could affect them deeply. One PHN described a situation with a suicidal young person:^17^ ‘*It’s not easy. If we had ready-made solutions, it would be, but we don’t. I probably didn’t tackle things the right way (…) It’s a feeling of panic you get in serious cases*’.

They spoke about their personal responsibility to protect others. This could create value conflicts between respecting the other person’s autonomy and their right to indulge in risky behaviour and protecting them from harm.^17,33^ Their front-line role meant experiencing ethical dilemmas and uncertainties. In Oberle and Tenove’s^34^ study, PHNs frequently questioned what they should do in complex cases, especially when they were asked by service users not to share information with other professionals. As one PHN said:^34^ ‘…*we’re always wrestling with that whole idea of “now whose rights are highest here? Gee, a doctor should know this,” but, on the other hand, they’d asked me not to tell’*. Dealing with conflicting loyalties and prioritizing was also found by Dahl et al.,^32^ where low resources combined with high expectations resulted in ethical dilemmas. Multiple client levels caused tensions and value conflicts, forcing PHNs to choose between providing care to more people with fewer needs and fewer with greater needs:^34^ ‘*Who should be in focus (individual, family or community)*?’ PHNs also experienced conflicting loyalties when they lacked resources and had to choose between spending time with pupils who were at risk but resisted change and spending time preventing illness and promoting the health of all the pupils.^33^ They were uncertain about whom to prioritize. One PHN said:^30^ ‘*Each one of us has over 500 pupils on our lists*’. The nurses found it important to provide the right help at the right time:^35^ ‘…*It is here and now and even earlier that it should be invested to promote good health*’. Prioritizing immediate action to protect a child could result in long-term estrangement from the family, leaving the PHN feeling powerless at no longer being able to help.^32^

PHNs spoke of uncertainties when performing certain tasks. Recording sensitive issues in schoolchildren’s medical records could cause worries, expressed as:^29^ ‘*Having to do one’s duty and being afraid to do wrong*’. This was particularly relevant when it involved mental illness, abuse or vulnerable children. The study by Laholt et al.^31^ revealed that visual technologies created new challenges for PHNs, as illustrated here:^31^ ‘*Once an adolescent came to my office, sharing a film he had taken at home of his drunken stepfather. That was challenging, especially because he thought the film was funny*’.

PHNs felt moral distress when service users received inferior care or were not treated with dignity and respect by healthcare providers.^28,34^ The uncertainty of outcomes for immigrant families caused a heightened sense of responsibility, especially related to the best interests of the child. One PHN used the words^28^ ‘*heart-breaking and worrying*’ to describe their job*.* PHNs also used the words^33^ ‘*very painful*’ to describe their own vulnerability. It was difficult to understand and defend how the system treated vulnerable families.^28,18,32^ In Kvamme and Voldner’s study,^28^ PHNs played a significant role in the lives of undocumented migrant families. One PHN mentioned an immigrant mother’s dilemma:^28^ ‘*It’s a tiny little child. Should she be obedient to the Norwegian authorities and move back to that country? In which case, should she bring her child or not bring her child? It is such a horrible dilemma that it is not easy for us to give any advice…’*

### Lack of boundaries – ‘At what point do you draw the line?’

The lack of boundaries in PHNs’ responsibility was evident in the studies.^17,28-32,34,35^ Clancy and Svensson^17^ described boundaries in responsibility as^17^ ‘*a corridor of half-open doors, providing openings and opportunities allowing for choices, and adding to uncertainties’*. The lack of rigid boundaries could leave each nurse feeling inadequate and alone. One said:^17^ ‘*It’s not written down anywhere how much responsibility we have. That’s just the way it is, we take it upon ourselves*’.

Boundaries were mentioned regarding where to draw the line between PHNs’ professional and private lives,^17,18,32,34^ between their relational and professional involvement in certain cases^17,28,29,18,34^ and between empowering clients or increasing their dependency on the nurse.^28,34,35^ PHNs wished to provide low threshold services for pupils. One school nurse in Hilli and Pedersen’s^35^ study stated: ‘*It is always open here – they may come anytime – they may come early in the morning or on their way home…They can always come…If I am here, I am here for the children…*’. The PHNs in the study by Tollefsen et al.^18^ described the uncertainty of never knowing who could turn up at the school nurse’s office, expressed in Clancy and Svensson’s^17^ study as ‘*The door to the other is always ajar*’.

PHNs spoke of the complex nature of their work and the difficulty in creating relational boundaries for involvement with service users,^17,28-30,18,33-35^ expressed by a nurse in Duncan’s^33^ study as ‘*…where is the beginning and the end of such responsibility?*’.

Teachers, school management and parents expected PHNs to have solutions to complex problems. As one school nurse said, much was expected of them:^30^ ‘*They are very happy to pass the ball to whomever catches it, in a way. When the kid isn’t as expected ‘Can someone fix this kid?’*. The burden of responsibility was often exacerbated by unclear boundaries in their role and duties. They often had to deal with situations that should have been addressed by other professionals.^28,30,31,35^ One nurse in Hilli and Pedersen’s^35^ study said: ‘*The greatest challenge is to reach over all children who don’t feel well, those sitting at home, children who for some reason don’t go to school…this is a big challenge for the school*’. One nurse in the study by Heggestad et al.^30^ summed it up as follows: *‘We are supposed to handle all the difficult things. Both the shameful things, and the emotional things, and those things that are uncomfortable for others to handle. Those are just thought of as “school nurse can handle that*”’. This resulted in temporary solutions due to limited time, ability and resources to get to the root of complex problems.^30^ A PHN in Oberle and Tenove’s^34^ study said: *‘Where my [problem] comes in is there just isn’t enough of me to do the kind of job to meet the need that’s there, and you know at what point do you draw the line and say, ‘“I* *have to stop here, I can’t do any more”*’.

### Feeling alone – ‘You have to do the job yourself; there is no one to take your place’

PHNs are described as independent professionals who often work alone.^17,29-33,35^ This independence is associated with ‘*… a degree of isolation*’.^33^ PHNs were interpreted by colleagues as ‘*“lonely birds” with a lack of role clarity*’.^30^ They often felt alone with their worries and responsibilities^17,29-32,35^ in risky situations^34^ and needed confirmation when making complex decisions.^30^ They felt vulnerable, as one PHN in the study by Tollefsen et al.^18^ said: ‘*I’ve been working alone for long periods. This has been tough because I had to make all the decisions on my own. I have no team to lean on*’. Another nurse reported feeling left alone with responsibility for clients in complex cases, being neither a psychologist nor a psychiatrist:^18^ ‘*I am only a public health nurse*’. Feeling alone with a strong sense of personal commitment could intensify PHNs’ feelings of ethical responsibility. Combined with the unpredictable nature of their work, this caused uncertainties and ethical deliberations.^18^ One PHN said:^17^ ‘*You have to do the job yourself; there is no one to take your place*’.

PHNs felt an obligation to collaborate.^34^ Referring clients to other services was a way of dealing with conflicting loyalties^33^ and a heavy workload.^30^ When they found collegial support, feelings of inadequacy became easier to bear.^17,30-32,35^ Collaborating with other professionals did not necessarily resolve conflicts,^33^ and could create new ethical dilemmas, due to, for example, teachers’ lack of interest in improving pupils’ health,^35^ power hierarchies involving doctors^18,34^ or information sharing and client confidentiality.^28-31,33-35^ PHNs felt responsible for facilitating collaboration^17,34^ and problem solving and had to manoeuvre among laws and ethics, needing confirmation and support.^30,31^

### Courage and commitment – ‘I know where my loyalty is: it’s with my clients’

Burdens of responsibility could cause worries, but also make PHNs feel needed and increase their job satisfaction.^17^ Tollefsen et al.^18^ described a PHN’s relationship with adolescents: ‘*It’s often when they’re at their most vulnerable… things they can’t tell anyone else, they share with me*’. They were proud of supporting their clients and enjoyed the challenges of their unpredictable work.^17,18,32^

Personal satisfaction was associated with showing respect, being true to their values and being able to uphold professional standards.^17,28,18,32,35^ Nurses spoke about the importance of promoting health and maintaining supportive relationships.^28,31,34,35^ They felt proud of being able to make a difference in people’s lives.^17,28,18^ Defending children’s rights and being strongly committed to supporting and empowering adolescents was a driving force for PHNs.^17,28,32,35^ Ethical responsibility involved ‘*standing up to fight*’,^17^ especially for children and adolescents.^28,31-32,34,35^

Making decisions in ethically demanding situations required courage and often came at a cost. PHNs mobilized the courage to speak their minds,^32^ even when they might lose the parents’ trust by doing so.^17,32,33,35^ They took responsibility in situations that called for action.^17,28,29,18,32^ They mentioned situations that required professional deliberation and courage, such as documenting suspicions of abuse.^29^ A PHN in the study by Laholt et al.^31^ described value conflicts, including a specific situation where a young girl wanted to commit suicide and the nurse told the parents and other professionals. The ethical dilemma of life and death justified the breach of confidentiality, despite damaging the nurse’s relationship to the girl. The nurse said:^31^ *‘She never came back to my office again’.*

The PHNs’ loyalty was with their clients^17,28,29,31,32,33,35^ even if it meant bending the rules.^34^ A nurse in Clancy and Svensson’s^17^ study said: ‘*One thing is for sure, I know where my loyalty is: it’s with my clients. Sometimes I really stand up for them and if nobody listens, then I become difficult*’.

## Discussion

### Overall interpretation of the findings – ‘Chivalrous knights in moral armour’

PHNs can be described as chivalrous crusaders willing to fight for their cause to help those in need. Like medieval knights, they are courageous, and bound by a moral code to do good and protect others. However, unlike knights of yore who had armies, these nurses often feel alone in battle, and they do not have masks to hide behind or heavy armour to protect them. Their battlefield knows no boundaries. Their moral armour emboldens them to stand up and fight to protect others yet leaves them exposed. Stories of medieval knights do not elaborate on the fact that even though their armour gave them courage, it was heavy and cumbersome to wear.

A knight's pledge of allegiance was to his lord, while PHNs answer to many. They are responsible for individuals and communities and work on the front line, close to their clients. PHNs see and witness children at risk of abuse, adolescents fighting mental health issues, immigrant families threatened with deportation, and youth in danger of committing suicide. They provide emotional support and have to deal with: ‘*the shameful things, and the emotional things, and those things that are uncomfortable for others to handle*’.^30^ Witnessing people in need implies a responsibility to help.^36^

PHNs feel the weight of moral responsibility and need help to bear their burdens. Unlike medieval knights, they do not have squires to alleviate their burdens. They wrestle with worry and uncertainties and use their knowledge and experience as shields and weapons. Knowledge, experience and collegial support can help, but are sometimes inadequate to negotiate complex ethical problems. Driven by their personal and professional values, they fight lonely battles clad in moral armour.

PHNs are described as ‘*lonely birds*’ *with a* ‘*lack of role clarity*’.^30^ They spoke of not knowing where to draw the line in relationships, between their personal and private lives or in referring difficult cases to other professionals. The very nature of ethical responsibility involves uncertainty and accountability. Levinas^12^ reminds us that we are vulnerable and cannot control our moral responsibility. It is greater than us. It precedes and immerses us and is something we have to live with.

PHNs appreciated their freedom to prioritize tasks. Freedom is, however, always curtailed and complicated. Moral freedom is a paradox. Seemingly, the greater the freedom, the greater the responsibility. According to Wallinvirta,^16^ freedom is a core element of moral responsibility. We have freedom to act morally but must take responsibility for our actions.^16^ Moral responsibility implies being trapped and free at the same time.^12^ PHNs are free to choose a course of action yet lack boundaries for their responsibilities and feel obliged to make the right choice. They experience this responsibility as personal, causing loneliness in difficult cases. Collaborative relationships help but do not always resolve their feeling of solitude. We are existentially alone^37^ and will always be confronted with new decisions.^15^

PHNs’ huge workload and low threshold services made prioritization difficult and sometimes forced them to compromise on the quality of their services. They often had little time and resources to perform their work, yet enough time to dwell on the health and well-being of their clients after being confronted with their needs. Relationships can make a lasting impression on us. These nurses worked closely with children and families. Proximity in moral responsibility is not about physical closeness.^38^ In Levinasian terms, *the trace of the other* leaves its mark, despite the other no longer being present.^37^ The nature of the nurses’ health promotion and prevention work with children, young people and families means that they follow families for years. PHNs do not work shifts or have handover reports to relieve them of their responsibility when their working hours end. The PHNs in the sample looked back at their decisions and had to live with them, and worried about possible outcomes, based on their knowledge and experience. To quote Levinas, they seemed to:^37^ ‘*Let the future and the most far-off things be the rule for the present day*’.

They experienced uncertainty and value conflicts when others were treated unethically. Their open-door policy in schools left them feeling vulnerable as they could never be fully prepared for new situations. Vulnerability can be interpreted as lacking strength, yet it is also associated with sensitivity, an awareness of one’s own feelings and compassion towards others.^39^ The nurses’ sensitivity gave them strength and enabled them to respond to the needs of others. According to Numminen et al.,^40^ ethical sensitivity in responsive relationships is a precursor to moral courage. The nurses’ loyalty was always with those in need. They felt acknowledged by their service users and took pride in their work. Recognition for their work can give PHNs a sense of self-respect and dignity which enables them to act as moral agents.^8^

These nurses had a clear sense of right and wrong and often responded with moral indignation and courage when their clients were not treated with respect. In addition to freedom, Wallinvirta^16^ also mentions love and guilt as core elements of responsibility. The PHNs do not specifically mention love or guilt. However, their compassion is evident in their narrations, such as their indignation when vulnerable others suffer. According to Løgstrup,^15^ compassion, and indignation are sovereign expressions of life. When human life and dignity are threatened, the ethical demand^15^ or appeal to respond^12^ emerges. Yet the ethical demand can never be fully met.^15^ We are not angels; and must do our best to live together responsibly.^17^ Despite feeling alone and inadequate, the nurses felt satisfaction and were willing to stand up and fight to ensure that others received adequate care. Courage enables nurses to face challenges and disapproval when doing what they believe is morally right.^40^ Courage is described by Numminen et al.^40^ as an attribute of a good nurse. It is interpreted as a reflexive response to unethical situations that threatened the health and well-being of vulnerable others.

### Discussion of method, strengths and limitations

All authors are PHNs. The first author is a PHN and senior researcher with experience in meta-synthesis research. The fourth author is a PHN, associate professor, and university lecturer in public health nursing. The remaining authors are experienced PHNs and university lecturers. All four contributed to the team with their unique perspectives. Their knowledge of the field through research and practice over 40 years was an asset, enabling critical discussions on interpretations of the findings. To ensure rigour, the authors also discussed the methodology and findings with meta-synthesis researchers who were not PHNs. The authors have followed the journal's research ethical guidelines,^41^ strived for transparency and provided details of all phases of the review process. A meta-summary is included to present the characteristics of each study.^23^ A supplementary file that illustrates the process of analysis has been submitted.

It can be argued that the review is not exhaustive and that Scandinavian countries are overrepresented. However, exhaustive sampling is not the only form of legitimate sampling for a research synthesis; a purposeful selection of information-rich studies can be sufficient for an in-depth synthesis.^42^ The number of reviewed studies is small, and the study is limited to PHNs working with children, young people and families. According to Noblit and Hare,^20^ a meta-ethnography is suitable for synthesizing even a small number of studies. It can also be argued that the findings overlap. However, this illustrates connectivity between the themes. The studies were published from 1992 to 2021 and illustrate reciprocal findings across studies for that period. It can also be considered a disadvantage that the authors come from a similar cultural background and that the included studies represent a narrow cultural context. The authors have created the overriding metaphor ‘chivalrous knights in moral armour’ based on their world view. Authors from other cultures would possibly have used other metaphors to illustrate their understanding of PHNs’ everyday heroism. However, the authors purport that the study has cross-cultural relevance. The findings give insight into PHNs experiences of ethical responsibility and their personal vulnerability when they act as moral agents and advocate for vulnerable others.

### Theoretical and clinical implications

The purpose of this study was to gain a deeper understanding of how PHNs experience ethical responsibility. This research is a key step towards developing a theory of ethical responsibility in public health nursing. The findings show that Clancy and Svensson’s^17^ descriptions of the dimensions of responsibility described as: *personal responsibility, boundaries, temporality, being alone, worry, fear and uncertainty, and a sense of satisfaction* have continued relevance. Wallinvirta’s^16^ concept of freedom*,* and Numminen et als.,^40^ deliberations on courage deepen our understanding of the phenomenon of ethical responsibility in public health nursing.

Ethical responsibility entails response to ethically challenging situations. The PHNs’ ethical responsibility determines their response and their clinical judgement helps guide their involvement. There are, however, limits to nurses’ knowledge and involvement in others’ lives. The findings are especially relevant for public health nursing education. Student PHNs need to understand that public health nursing involves satisfaction but also uncertainties, worries and loneliness, and that these feelings are often adequate responses to ethically challenging situations. They are indicative of PHNs’ professional dignity, humanness and vulnerability; not a sign of weakness but a strength and necessary precursor to courage and commitment. The authors recommend further studies of ethical responsibility in public health nursing across the life span and in other nursing specialities. Such studies would enhance knowledge of nurses’ clinical reflections and ethical responses to the challenges they face in their work.

## Conclusion

This meta-ethnography has shown PHNs’ dedication and vulnerability in response to the ethical demand to protect vulnerable others. It also illustrates the paradoxical nature of freedom, and how freedom, due to a lack of boundaries, heightens the nurses’ sense of responsibility. Their ethical sensitivity and respect for their service users give them the courage to act morally. Nurses are required to use research-based evidence in their practice. The study has shown that what is evident in a situation may necessitate ethical reflection that is not easy to articulate and not necessarily covered by generalized scientific research. The research has also provided insight into the asymmetrical and all-encompassing nature of ethical responsibility. This occurs in situations when the public health nurse, despite her formal position of power, feels trapped and vulnerable when she acts as a moral agent and advocate for vulnerable others.
